# Identification of an Immune Gene Expression Signature for Predicting Lung Squamous Cell Carcinoma Prognosis

**DOI:** 10.1155/2020/5024942

**Published:** 2020-06-27

**Authors:** Yubo Yan, Minghui Zhang, Shanqi Xu, Shidong Xu

**Affiliations:** ^1^Department of Thoracic Surgery, Harbin Medical University Cancer Hospital, Harbin, China; ^2^Department of Medical Oncology, Harbin Medical University Cancer Hospital, Harbin, China

## Abstract

Growing evidence indicates that immune-related biomarkers play an important role in tumor processes. This study investigates immune-related gene expression and its prognostic value in lung squamous cell carcinoma (LUSC). A cohort of 493 samples of patients with LUSC was collected and analyzed from data generated by the TCGA Research Network and ImmPort database. The R coxph package was employed to mine significant immune-related genes using univariate analysis. Lasso and stepwise regression analyses were used to construct the LUSC prognosis prediction model, and clusterProfiler was used for gene functional annotation and enrichment analysis. The Kaplan-Meier analysis and ROC were used to evaluate the model efficiency in predicting and classifying LUSC case prognoses. We identified 14 immune-related genes to incorporate into our prognosis model. The patients were divided into two subgroups (Risk-H and Risk-L) according to their risk score values. Compared to Risk-L patients, Risk-H patients showed significantly improved overall survival (OS) in both training and testing sets. Functional annotation indicated that the 14 identified genes were mainly enriched in several immune-related pathways. Our results also revealed that a risk score value was correlated with various signaling pathways, such as the JAK-STA signaling pathway. Establishment of a nomogram for clinical application demonstrated that our immune-related model exhibited good predictive prognostic performance. Our predictive prognosis model based on immune signatures has potential clinical implications for assessing the overall survival and precise treatment for patients with LUSC.

## 1. Introduction

Lung cancer remains the leading cause of cancer incidence and mortality worldwide [1]. Non-small cell lung cancer (NSCLC) is the most common type of lung cancer and is classified into two major histological subtypes, lung adenocarcinoma (LUAD) and lung squamous cell carcinoma (LUSC), each with distinct genomic and immunological profiles [2]. The discovery of epidermal growth factor receptor (EGFR), anaplastic lymphoma kinase (ALK), and ROS proto-oncogene 1 (ROS1) gene targets and the development of corresponding target drugs have prolonged the survival of patients with NSCLC [3]. Currently, progress has been slow in the development of LUSC treatments due to the lack of effective targets; however, continuous developments in immunotherapy have provided a new direction for LUSC treatment [4]. Immunocyte infiltration, which is speculated to represent the active tumor response, can be detected among most solid tumors in humans; specifically, lymphocyte infiltration in LUSC has certain survival benefits [5]. Therefore, understanding the immune gene signatures of LUSC is highly significant as it could have predictive prognosis implications.

At present, the tumor-node-metastasis (TNM) classification system has been recognized as the most meaningful indicator for prognosis and can inform therapeutic decisions for LUAD as well as LUSC treatment [6]. Nonetheless, this classification system is imprecise because various progression levels and overall survival (OS) results can be observed among cases in the same stage. Therefore, novel markers are urgently needed to recognize patients with high recurrence risk. A precisely indicated prognosis significantly affects a clinician's decision to recommend adjuvant therapy. Additionally, there is increasing need to improve prognosis prediction tools.

Biomarkers can reliably predict disease prognosis as well as patient survival. As a result, they are meaningful in the decision-making process for clinical LUSC treatment. In recent years, an increasing number of articles have recommended that gene expression profiles can be applied to predict and stratify the survival prognosis of LUSC cases [7, 8]. However, the role of immune-related genes in LUSC is unclear. Therefore, openly accessible large databases that contain gene expression profiles allow us to mine creditable biomarkers for predicting and classifying LUSC prognosis.

This study aimed at establishing and verifying a prognosis prediction model for LUSC based on genes related to immunity and patient clinical features derived from the Cancer Genome Atlas (TCGA) Research Network and ImmPort database.

## 2. Materials and Methods

### 2.1. Data Collection

Gene expression and clinical LUSC patient data were downloaded from the TCGA Research Network (https://www.cancer.gov/tcga), and the gene set related to immunity was obtained from the ImmPort database (https://www.immport.org). The raw data were preprocessed as follows: (1) samples without clinical data were removed; (2) normal tissue sample data were removed; (3) genes with fragments per kilobase per million reads (FPKM) values of 0 in more than half the samples were removed; and (4) the expression profiles of immune-related genes were saved. After preprocessing, 493 samples comprising 1421 immune-related genes were utilized for further model analysis. The 493 samples were randomized into training and test sets. All samples underwent 500 iterations of random grouping with replacement to eliminate the impact of random allocation bias on model stability. Data in the training (*n* = 245) and test (*n* = 248) sets are presented in Table 1. There was no statistically significant difference between the two sets, which indicated reasonable sample grouping.

### 2.2. Prognostic Signature

The correlation of immune-related gene expression with patient OS was assessed through the univariate Cox proportional hazards regression analysis using the survival coxph function of the R package. Genes with *p* values < 0.05 were identified as candidate genes. Subsequently, the number of candidate genes was reduced according to the least absolute shrinkage and selection operator lasso-Cox method using the glmnet and MASS function of the R package. Genes most significantly related to immunity were selected to construct the prognosis risk score model. The risk score model was formulated as follows:

(1) $$\begin{array}{c} \text{Risk score}=\overset{n}{\underset{i=0}{\sum }}\beta i\times Xi, \end{array}$$

where *βi* represents the coefficient of every gene and *χi* stands for gene expression level (FPKM). The median risk score value was the threshold for classifying samples into high-risk (Risk-H) or low-risk (Risk-L) groups. ROC and the Kaplan-Meier (KM) analyses were carried out to evaluate model efficiency, stability, and accuracy in predicting and classifying LUSC case prognoses.

### 2.3. Functional Annotations

Eventually, 14 genes were selected and their gene families annotated according to human gene classification within the HUGO Gene Nomenclature (HGNC) database. The R package clusterProfiler was employed to carry out enrichment analysis on the 14 screened genes related to immunity and specific to prognosis. The KEGG enrichment analysis score was evaluated using the ssGSEA function of the R package GSVA [9]. Association with the risk score value was also calculated. Clustering analysis was then carried out according to the pathway enrichment score for each sample.

### 2.4. Association between Risk Score Value and Clinical Features

Associations between relevant clinical factors (such as stage (T, N, or M), subdivision, age, and smoking habit) and risk score value were analyzed. Then, a nomogram model was constructed, and a forest plot was drawn according to relevant clinical features and risk score values. The associations between risk score value and clinical features related to patient survival were also analyzed.

### 2.5. Statistical Analyses

Independent subgroups were analyzed using the Chi-square test or Fisher's exact test. Univariate and multivariate analyses were performed using the Cox regression. Differences in OS between high- and low-risk groups were evaluated according to the Kaplan-Meier survival curve. The sensitivity and specificity of the diagnosis and prognosis prediction model were determined and assessed using the ROC area under the curve (AUC). The Kruskal-Wallis test was used to evaluate the relationships of risk score with different clinical factors. A two-tailed *p* value of < 0.05 was recognized as statistically significant. Statistical analyses were performed using the R software (Version 3.5.5; R Core Team, 2016).

## 3. Results

### 3.1. Data Processing

**Figure 1:**
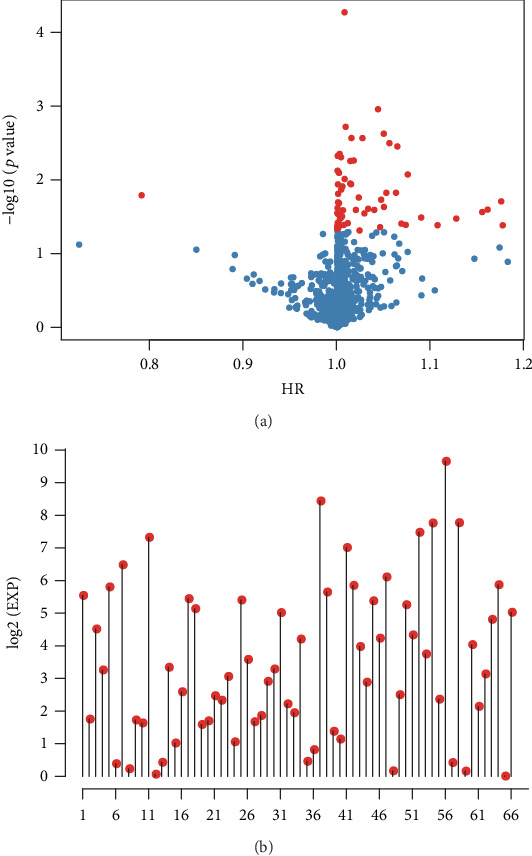
Differential gene expression in lung squamous cell carcinoma. (a) The relationships of the -log10 (*p* values) and HR. (b) The expression levels of 66 differentially expressed genes. Red dots represent significantly different immune-related genes (*p* < 0.05) regarding prognosis.

Sixty-six immune-related, prognosis-specific genes were mined. The *p* value relationships of the 66 genes with hazard ratios (HRs) and expression levels are displayed in Figure 1.

### 3.2. Establishment of the Prognosis Prediction Model

**Figure 2:**
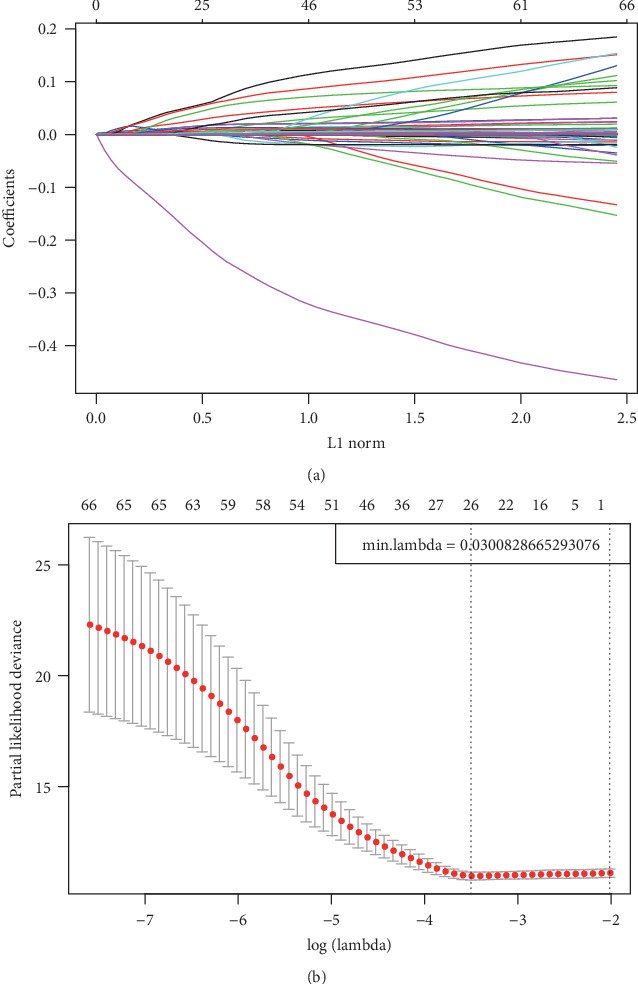
Construction of the prognosis prediction model for LUSC patients by LASSO. (a) The changing trajectory of each independent variable. The horizontal axis represents the log value of the independent variable lambda, and the vertical axis represents the coefficient of the independent variable. (b) Confidence intervals for each lambda.

Sixty-six immune-related genes were identified, although that number was inappropriately high for use in clinical detection. Therefore, the scope of genes related to immunity was narrowed to maintain high accuracy. The 66 genes were compressed through lasso regression to reduce the number of genes incorporated in the risk model. The variation trajectories for all independent variables (Figure 2(a)) suggested that the coefficients of a larger number of independent parameters were close to 0 as lambda gradually increased. The confidence interval (CI) under every lambda (Figure 2(b)) revealed that the best model was obtained at a lambda value of 0.03, which was consequently chosen for the eventual model that included 26 immunity-related genes. In addition, the MASS of the R package was utilized in stepwise regression analysis based on Akaike data criteria to obtain 14 genes used to construct the risk model.

**Figure 3:**
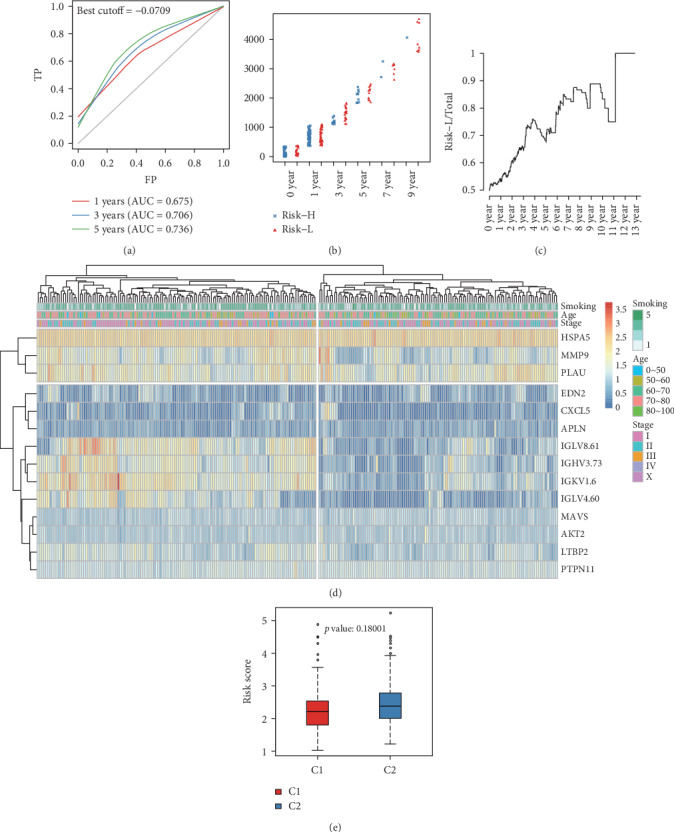
Verification of the stability of the prognosis prediction model for patients with lung squamous cell carcinoma in the training cohort. (a) Survival predicted ROC curves for the training cohort. (b) Distribution of samples in Risk-H and Risk-L groups of the training cohort divided by different OS. (c) The proportion of low-risk samples in total samples varies with OS. (d) Clustering results of the training cohort. (e) Differences in risk score values between Risk-H and Risk-L groups clustered by gene expression in the training cohort.

Each sample from the training cohort was then incorporated into the formula for calculating the risk score value. The OS for all samples is shown in Figure S1. Analysis of the model efficiency in predicting the 1-5-year OS resulted in a mean AUC value reaching 0.703 (Figure 3(a)). Sample distributions in Risk-H and Risk-L groups under different OS durations suggested that the 5-year sample size of the Risk-H group was reduced relative to that of Risk-L group (Figures 3(b) and 3(c)). The sample clustering results in the training cohort are presented in Figure 3(d). The 14 genes were clustered into high and low expression groups (Figure 3(e)). To verify the credibility of the prognosis prediction model, the expression profiles of the 14 genes were collected from the test cohort and incorporated into the verification model. The risk score values for the samples in the test cohort corresponded with those in the training cohort (Figure S2). To further verify model creditability and stability in prognosis prediction, the expression profiles of the 14 genes collected from 493 samples were incorporated into the model to calculate the risk score values. The results were consistent with the test set validation results (Figure S3). Taken together, the prognosis prediction model based on 14 immune-related gene expression profiles displayed superb stability and predictive accuracy in identifying immune-related characteristics.

**Figure 4:**
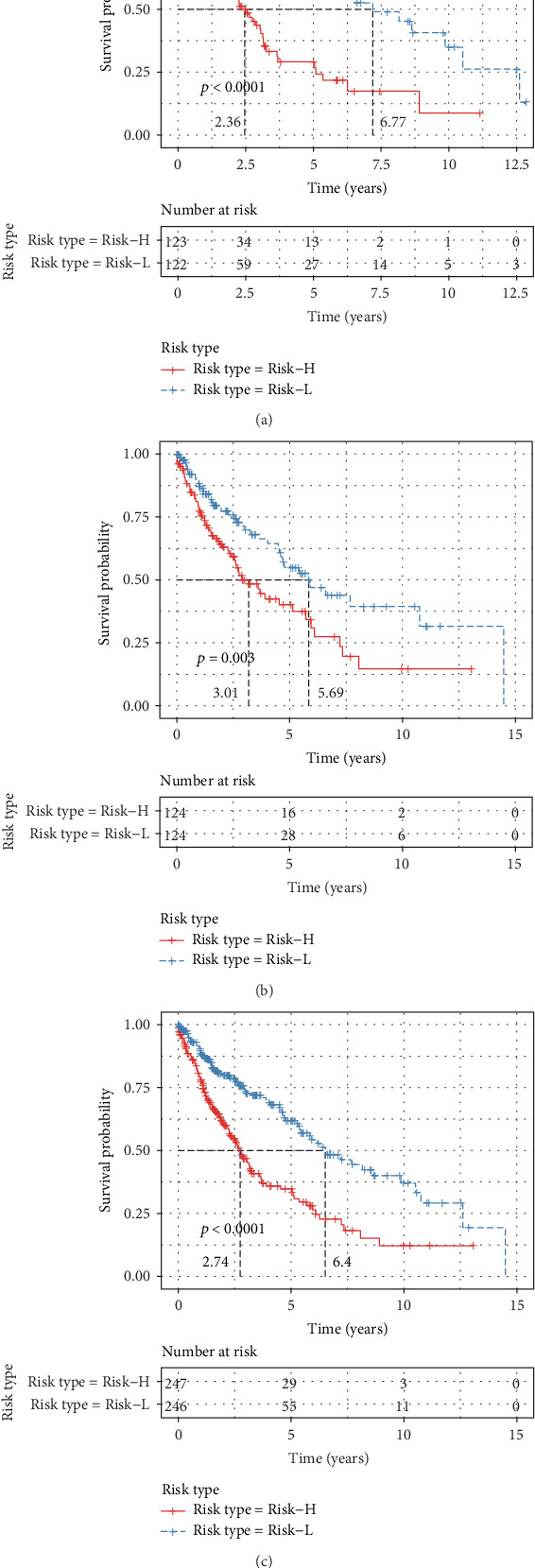
The Kaplan-Meier survival curve of the 14-gene risk model in predicting the Risk-H and Risk-L groups on the training set (a), testing set (b), and all samples (c).

KM survival curves were plotted for the risk model based on 14 genes in the Risk-H and Risk-L groups of the training cohort, test cohort, and the whole dataset (combined cohort). The KM survival curves of the training, test, and combined cohorts are displayed in Figure 4(a) (*p* < 0.001), Figure 4(b) (*p* = 0.003), and Figure 4(c) (*p* < 0.001), respectively.

### 3.3. Functional Annotation of Immunity-Related Genes

**Figure 5:**
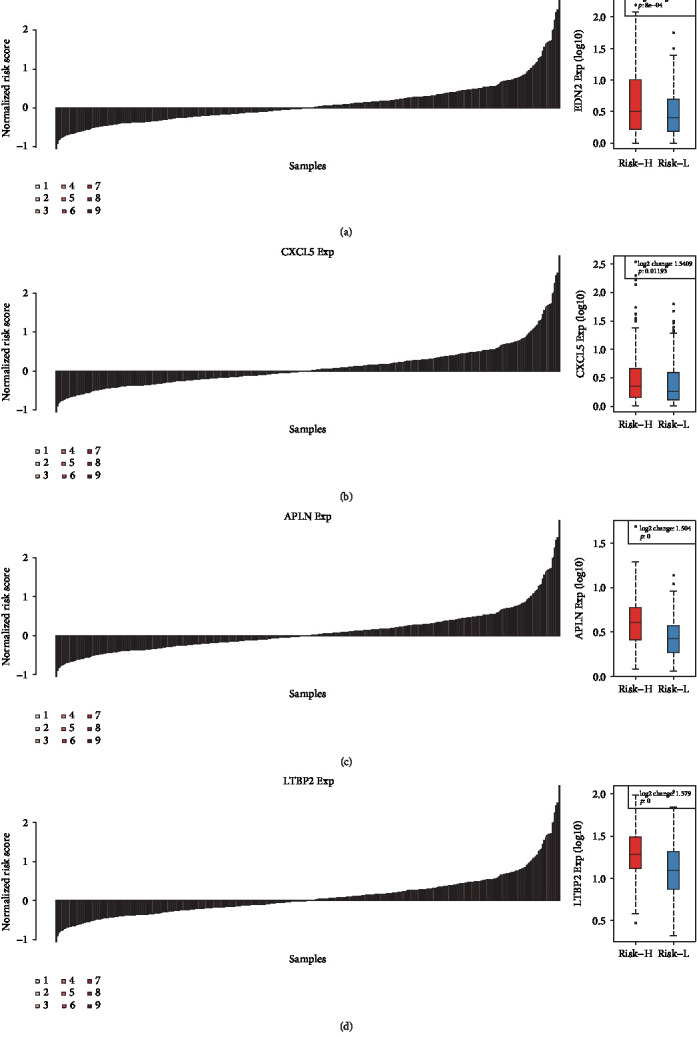
The expression differences of the EDN2 (a), CXCL5 (b), APLN (c), and LTBP2 (d) between the Risk-H and Risk-L groups.

The 14 gene families annotated based on human gene classification in the HGNC database (Table 2) were enriched in the endogenous ligands and latent transforming growth factor *β*-binding proteins (LTBP) gene families. Moreover, the expression levels of four genes (*END2*, *CXCL5*, *APLN*, and *LTBP2*) from these two gene families differed significantly between the Risk-H and Risk-L groups (Figure 5).

### 3.4. Association between Risk Score Value, Signal Pathways, and Sample Clinical Features

**Figure 6:**
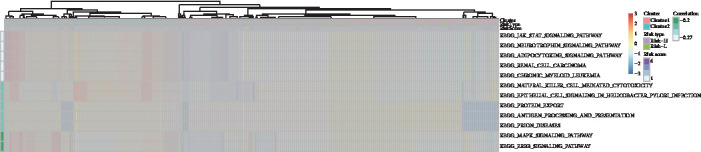
Correlation of risk score with signaling pathways.

The KEGG functional enrichment scores of all samples analyzed using the ssGSEA function of the R software GSVA package were correlated with risk score values and resulted in the acquisition of 41 relevant KEGG pathways. Cluster analysis was performed according to enrichment scores as shown in Figure 6. The most correlated pathway was the JAK/STAT signaling pathway.

The relationship between different clinical parameters (including stage (T, N, or M), gender, subdivision, age, and smoking habit) and risk score value was explored (Figure S4). The clinical features did not reveal a relationship with risk score value, except for age, indicating that risk score was relatively independent of the evaluated clinical characteristics.

### 3.5. Nomogram Prediction Model Establishment

**Figure 7:**
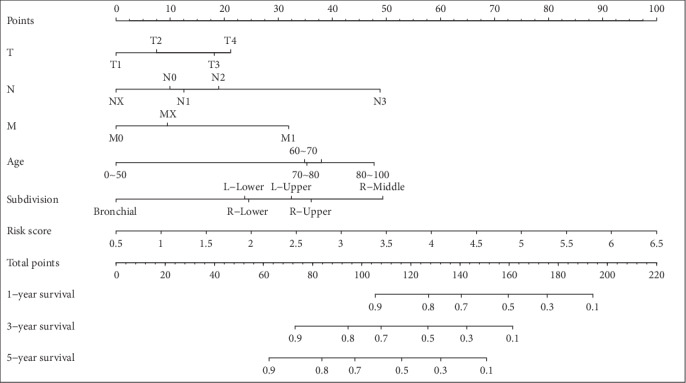
The nomogram model constructed by combining the stage-T, stage-N, stage-M, age, subdivision, and risk score.

**Figure 8:**
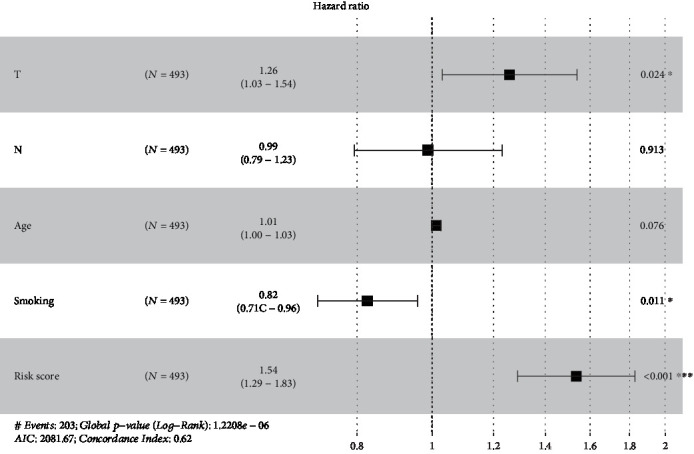
The forest plot constructed by combining the stage-T, stage-N, age, smoking, and risk score.

Risk score value was used in combination with clinical features to establish the nomogram model (Figure 7) in which risk score exhibited a pronounced association, with the greatest influence on survival rate prediction. This suggested that the risk model based on 14 genes displayed favorable performance in predicting the prognosis of LUSC. The forest plot based on risk score value and clinical features (Figure 8) indicated a risk score HR of 1.54 (*p* < 0.001).

## 4. Discussion

Our study developed a novel prognostic model employing 14 immune-related genes using data from the TCGA Research Network and ImmPort database. This prognostic model successfully predicted LUSC patient prognosis.

Surgical resection offers the most effective treatment for early-stage LUSC [10]. Adjuvant chemotherapy or EGFR-TKI improves the survival of stage II–III lung cancer patients after surgery [11, 12]. Therefore, adjuvant chemotherapy has been the standard care for resected stage II–III LUSC patients albeit many patients do not benefit from this form of chemotherapy. This phenomenon may be related to tumor heterogeneity. Our prediction model accurately identified early LUSC patients at high risk of recurrence.

The association between the immune system and pathogenesis, as well as the progression of malignancies, has drawn increasing attention in recent years. Unlike the rapid development of LUAD treatment strategies, LUSC treatment options have progressed more slowly. Recently, immune checkpoint inhibitors that target programmed cell death 1 (PD-1) and its ligand (PD-L1) have shifted the paradigm in LUSC treatment. To date, several anti-PD-1/PD-L1 antibodies have been approved for patients with advanced NSCLC [13–15]. Emerging evidence indicates that PD-L1 expression could predict anti-PD-1/PD-L1 therapy response in patients with NSCLC [16]. Inspiringly, the latest reports have demonstrated that gene profiling has the potential to predict patient response to immune checkpoint inhibitors [17–19]. In addition, the association of risk score value with relevant signal pathways was explored with JAK/STAT revealed as the most significantly correlated pathway. A previous study indicated that the JAK/STAT signaling pathway plays an important role in immunity regulation in the tumor microenvironment [20]. Given our results, drug-induced interference with the expression of this pathway may provide a new direction for LUSC treatment.

Distribution of the 14 immune-related genes was investigated in Risk-H and Risk-L samples. Seven of the 14 genes, including *PTPN11*, *MAVS*, *CXCL5*, *PLAU*, *MMP9*, *AKT2*, and *HSPA5*, reportedly participate in the pathological processes of the immune microenvironment, as well as the pathogenesis, malignant transformation, and progression of LUSC, which exhibited marked correlation with patient survival and prognosis [21–26]. Our results demonstrated that bioinformatics mining using available research is highly reliable and accurate. Nonetheless, the association between LUSC and *EDN2* and *LTBP2* genes, which may be enriched in the endogenous ligand and LTBP gene families, has not been verified in either basic or clinical studies. *EDN2* is reportedly involved in regulating malignant cancer cell proliferation and invasion, which can affect cytokine-mediated signaling pathways as well as modulate the activation and chemotaxis of immunocytes [27]. At the same time, *LTBP2* has been established as a prognostic marker for diverse cancer types and can control tumor cell sensitivity to immunotherapy [28, 29]. Elucidation of the roles of *END2* and *LTBP2* in NSCLC is currently underway in our laboratory.

There were several limitations of the present study. First, our study was based on data from public datasets without prospective testing. Second, of the immune-related genes used in the prognostic model, the roles of seven genes in NSCLC are unclear. Their prognostic value should be validated by other cohorts. Third, whether patients received immunotherapy is uncertain; therefore, the predictive value of the prognostic model for immunotherapy could not be directly evaluated.

## 5. Conclusions

We identified new prognostic markers for LUSC that contribute to classifying patients with LUSC based on their immune molecular subtypes. Our predictive prognosis model based on immune signatures has potential clinical implications for assessing the overall survival. These findings should be validated in prospective studies.

## Figures and Tables

**Table 1:** Patient characteristics with lung squamous cell carcinoma in training and testing sets.

Clinical features	Overall	Training set	Testing set	*p* value
OS	493	245	248	0.9383
Event	493	245	248	0.9293
Alive	284	140	144	
Dead	209	105	104	
T	493	245	248	0.4717
T1	114	49	65	
T2	286	146	140	
T3	70	40	30	
T4	23	10	13	
N	493	242	246	0.7437
N0	316	160	156	
N1	127	62	65	
N2	40	19	21	
N3	5	1	4	
NX	5	3	2	
M	493	208	204	0.6093
M0	405	206	199	
M1	7	2	5	
MX	81	37	44	
Stage	493	244	245	0.4364
I	241	116	125	
II	158	88	70	
III	83	38	45	
IV	7	2	5	
X	4	1	3	
Age	493	245	248	0.6387
0~50	22	9	13	
50~60	73	40	33	
60~70	181	85	96	
70~80	191	97	94	
80~100	26	14	12	
Subdivision	493	235	240	0.8473
Bronchial	10	5	5	
L-Lower	74	33	41	
L-Upper	135	68	67	
R-Lower	106	52	54	
R-Middle	18	10	8	
R-Upper	132	67	65	
Gender	493	245	248	0.9648
Female	128	63	65	
Male	365	182	183	

**Table 2:** Gene function annotation results.

Gene family	Genes	*p* value
Endogenous ligands	EDN2/CXCL5/APLN	0.0003
Latent transforming growth factor beta-binding proteins	LTBP2	0.0030
Heat shock 70 kDa proteins	HSPA5	0.0108
M10 matrix metallopeptidases	MMP9	0.0150
Caspase recruitment domain containing	MAVS	0.0185
SH2 domain containing	PTPN11	0.0599
Pleckstrin homology domain containing	AKT2	0.1180
Unknown	IGLV8.61/IGHV3.73/IGLV4.60/PLAU/IGKV1.6:	1

## Data Availability

The data used to support the findings of this study are included within the article.

## Abbreviations

NSCLC: Non-small cell lung cancer
LUAD: Lung adenocarcinoma
LUSC: Lung squamous cell carcinoma
EGFR: Epidermal growth factor receptor
ALK: Anaplastic lymphoma kinase
ROS1: ROS proto-oncogene 1
TNM: Tumor-node-metastasis
OS: Overall survival
TCGA: The Cancer Genome Atlas
AUC: Area under the curve
HRs: Hazard ratios
CI: Confidence interval
PD-1: Programmed cell death 1
PD-L1: Programmed cell death ligand-1.

## Supplementary Materials

Supplementary Materials Figure S1: distribution of overall survival in lung squamous cell carcinoma. Figure S2: verification of the reliability of the prognosis prediction model including 14 immune-related genes for LUSC patients in test set. Figure S3: verification of the stability of the prognosis prediction model including 14 immune-related genes for all the samples. Figure S4: the relationships of different clinical factors with risk score.
