# Supplementary material for: Identification of an Immune Gene Expression Signature for Predicting Lung Squamous Cell Carcinoma Prognosis
Source: Biomed Res Int. 2020 Jun 27;2020:5024942. doi: 10.1155/2020/5024942 (PMC7338973; doi:10.1155/2020/5024942)
Supplement: Supplementary Materials — Figure S1: distribution of overall survival in lung squamous cell carcinoma. Figure S2: verification of the reliability of the prognosis prediction model including 14 immune-related genes for LUSC patients in test set. Figure S3: verification of the stability of the prognosis prediction model including 14 immune-related genes for all the samples. Figure S4: the relationships of different clinical factors with risk score. [file 5024942.f1.pdf]

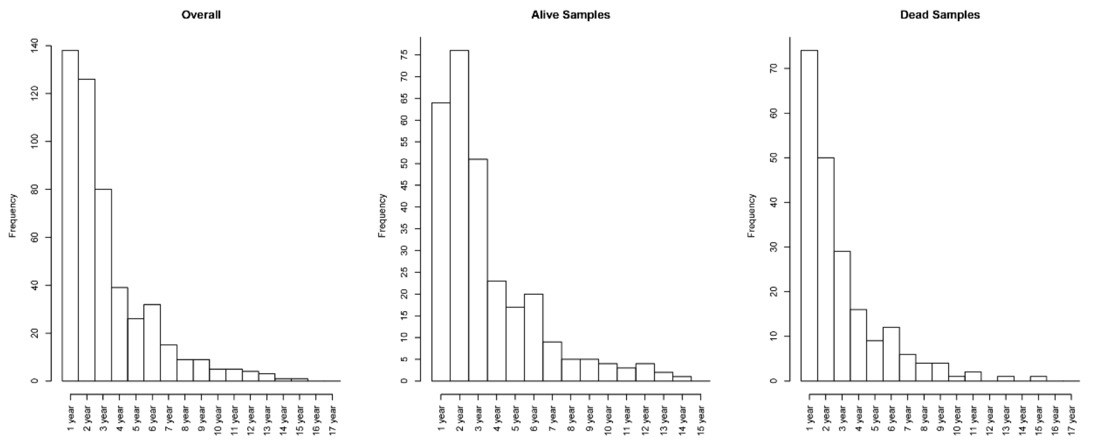

FIGURE S1 Distribution of overall survival in lung squamous cell carcinoma

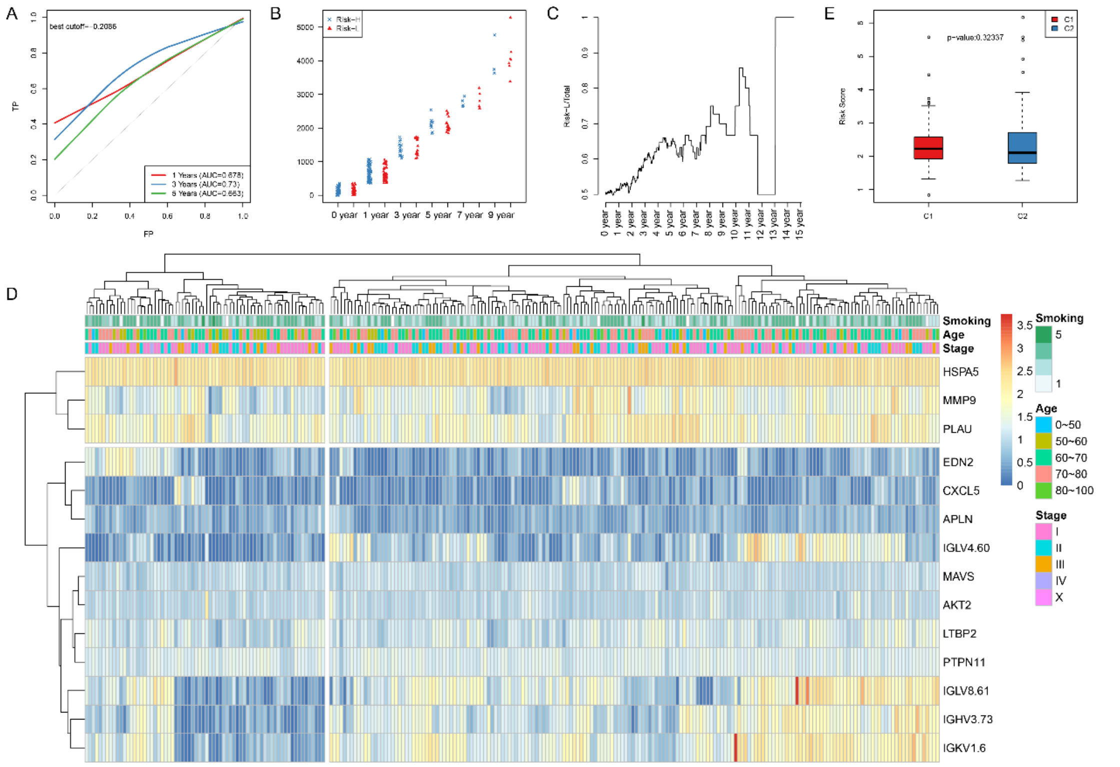

FIGURE S2 Verify the reliability of the prognosis prediction model included 14 immune-related genes for LUSC patients in test set. (A) The survival predicted ROC curves of 14-gene risk model in test set. (B) The distribution of samples in Risk-H and Risk-L groups of test set divided through 14-gene risk model under different OS. (C) The level of Risk-L group/Total sample size with the extension in OS in the test set. (D) The clustering results of test set samples. (E) Difference in the Riskscore between the two groups which had been clustered by the expression of 14 genes of test set samples.

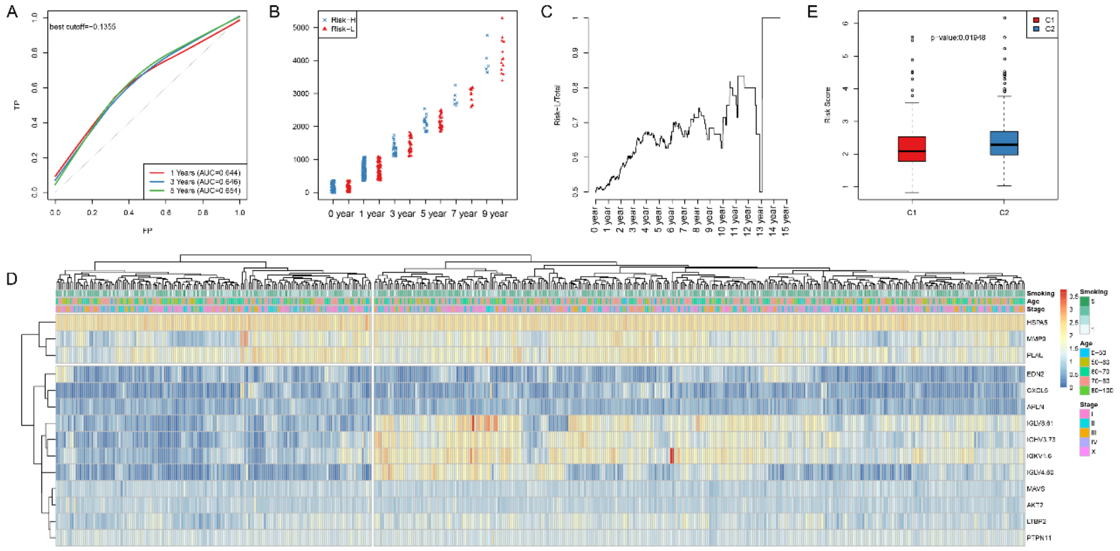

FIGURE S3 Verify the stability of the prognosis prediction model included 14 immune-related genes for all the samples. (A) The survival predicted ROC curves of 14-gene risk model. (B) The distribution of samples in Risk-H and Risk-L groups divided through 14-gene risk model under different OS. (C) The level of Risk-L group/Total sample size with the extension in OS. (D) The clustering results of all the samples. (E) Difference in the Riskscore between the two groups which had been clustered by the expression of 14 genes.

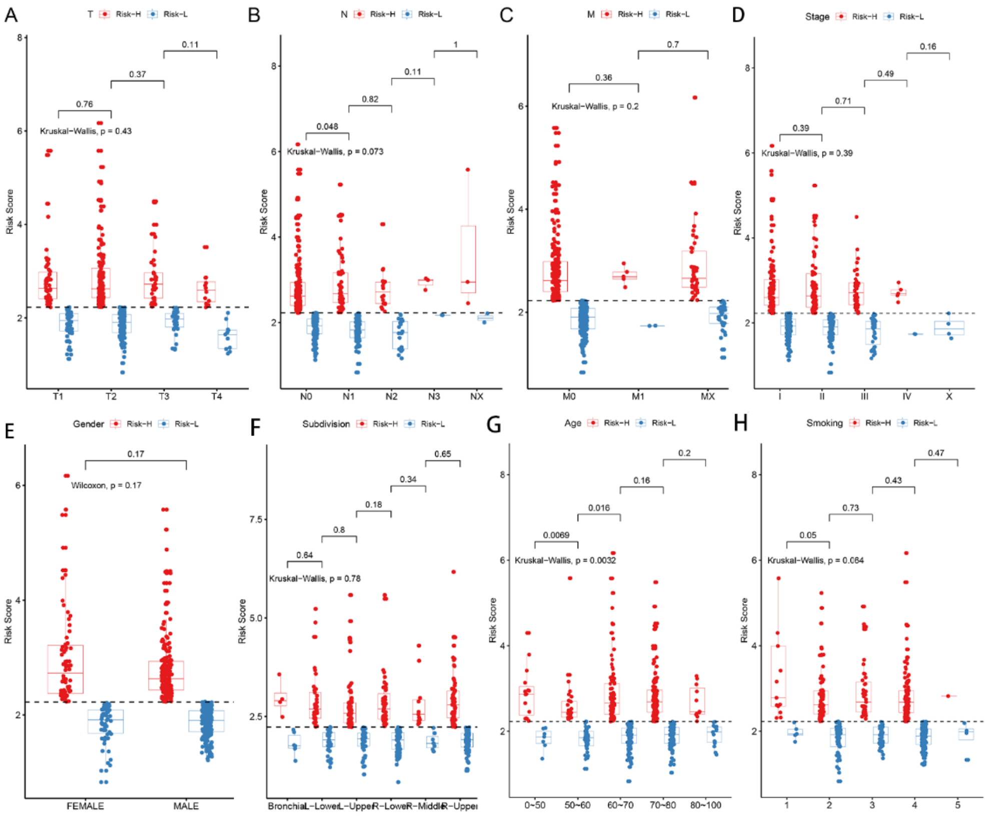

FIGURE S4 The relationships of different clinical factors with Riskscore. Comparison of Riskscore among different stage-T (A), stage-N (B) , stage-M (C), , stage (D) , gender (E) , subdivision (F) , age (G) and smoking (H).
